# Is identification and dissection of the external laryngeal nerve necessary during thyroidectomy? A prospective study

**DOI:** 10.1186/s12893-023-02287-x

**Published:** 2024-02-04

**Authors:** Soukayna Bourabaa, Abdellatif Settaf

**Affiliations:** Department of Surgery B, Ibn Sina Hospital, Faculty of Medicine and Pharmacy, Mohammed V University, Rabat, Morocco

**Keywords:** External branch of the superior laryngeal nerve, External laryngeal nerve, Thyroidectomy

## Abstract

**Background:**

Compared to the recurrent laryngeal nerve, the EBSLN (or external laryngeal nerve) is less studied in terms of its course and relationship with the thyroid gland. This is a prospective intraoperative study designed to identify the anatomical variations of the EBSLN in relation to the IPC, the superior thyroid pedicle, and the point where the nerve crosses the STA. Additionally, the study aims to propose a technical procedure for its preservation.

**Methods:**

We conducted a prospective study of 50 patients (total of 100 nerves) undergoing total thyroidectomy at the Department of Surgery ‘B’ in Ibn Sina Hospital, Rabat. Intraoperatively, the EBSLN was visually identified and preserved before ligating the superior thyroid vessels. Each nerve was categorized using established classification systems.

**Results:**

The overall pooled EBSLN identification rate was 82%. Cernea type IIa (nerves crossing the STA less than 1 cm above the upper edge of the superior thyroid pole) and Friedman type II (nerves piercing the lower fibers of the IPC) were the most prevalent (64% and 44%, respectively). Kierner type IV (nerves crossing the branches of the STA immediately above the upper pole of the thyroid gland) was represented in 27% of cases.

**Conclusion:**

A better understanding of surgical anatomy of the neck allows for better results of thyroidectomy by preserving the external and recurrent laryngeal nerves.

## Introduction

The external laryngeal nerve is the only motor nerve that supplies the cricothyroid muscle, which is the tensor muscle of the vocal folds and raises the pitch of the voice [1–5]. Because of the close anatomical relationship between the EBSLN and the thyroid gland [2–6], the nerve is especially in danger of inadvertent trauma during thyroid surgery [2, 7–9], inflammation and fibrosis such as in thyroiditis [10], malignancy, radiation damage or previous surgery [11], increases that risk. This can lead to long-term morbidity, particularly for woman and professional voice users such as singers and teachers and can harm their quality of life [12, 13].

Because of its anatomical variation, the EBSLN is injured in up to 58% of all thyroidectomies [9, 14–17], and there is no consensus regarding the best protocol for its preservation during surgery. Lack of EBSLN identification and subsequent preservation during surgery or inadequate knowledge of its variable anatomy are some of the factors thought to be responsible for the high incidence of its injury.

The clinical presentation can be pauci-symptomatic or asymptomatic, but may result in voice changes such as hoarseness, quick vocal fatigue, decreased high-pitched sounds, and rarely, dysphonia. The positive diagnosis is often more difficult than for recurrent nerve injury. Indeed, the vocal cords appear normal and laryngoscopy is often normal. However, two exams can help make the diagnosis; electromyography and laryngeal videostroboscopy.

Identifying and preserving any anatomical structure is the key issue in any surgical intervention. However, many surgeons tend to avoid exposing and identifying the EBSLN during thyroidectomy, prompting Delbridge [18] to declare it the ‘*neglected nerve in thyroid surgery*’.

Different authors have attempted to describe the course of the laryngeal nerve and its relationship with the superior thyroid vessels, and there are different surgical and anatomical classifications mentioned in the literature for the EBSLN.

## Methods

This is a prospective intraoperative study involving 50 patients who underwent total thyroidectomy for symptomatic goiters, compressive and plunging goiters, Basedow disease, and thyroid nodules suspected for malignancy, operated by the same surgeon at Ibn Sina Hospital in Rabat.

**Fig. 1:**
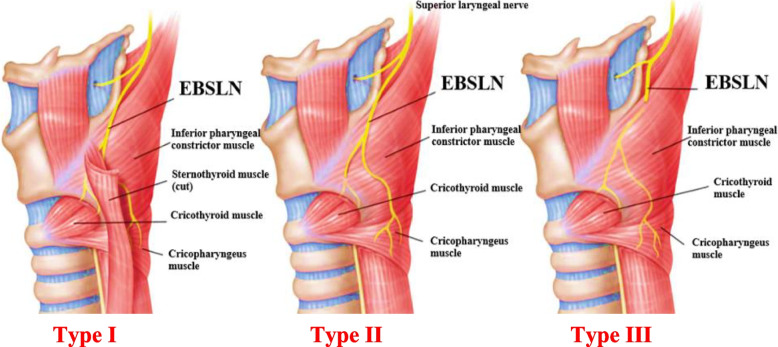
Friedman classification [3]. **Type I:** The EBSLN runs its whole course superficially or laterally to the IPC, descending with the superior thyroid vessels until it terminates in the CTM. **Type II:** the EBSLN penetrates the IPC about 1 cm proximal of the CTM. **Type III:** The EBSLN dives under the superior fibers of the IPC, remaining covered by this muscle throughout its course to the CTM

A systematic identification of the EBSLN was performed. The superior thyroid pedicle was approached first. The nerf was systematically searched for and dissected on both sides, first at the surface of the IPC, which it crosses at a variable level, allowing the nerve to be classified as per Friedman [3] anatomical classification (Fig. 1).

**Fig. 2:**
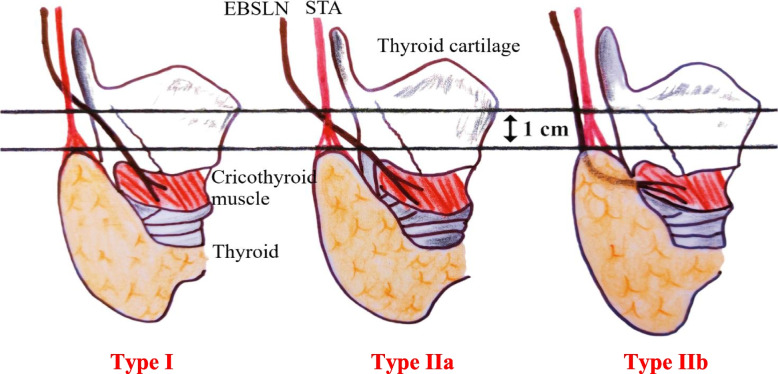
Cernea classification. **Type I:** The EBSLN crosses the STA more than 1 cm above the upper edge of the superior thyroid pole. **Type IIa:** The EBSLN crosses the STA less than 1 cm above the upper edge of the superior thyroid pole. **Type IIb: **The EBSLN crosses the STA below the upper edge of the superior thyroid pole

The nerve is located and carefully dissected during the approach to the superior thyroid vessels. The crossing of the SLN and the STA has been studied and given rise to several classifications, in particular Cernea classification [2] defined by the distance between this crossing and the upper pole of the thyroid gland (Fig. 2).

**Fig. 3:**
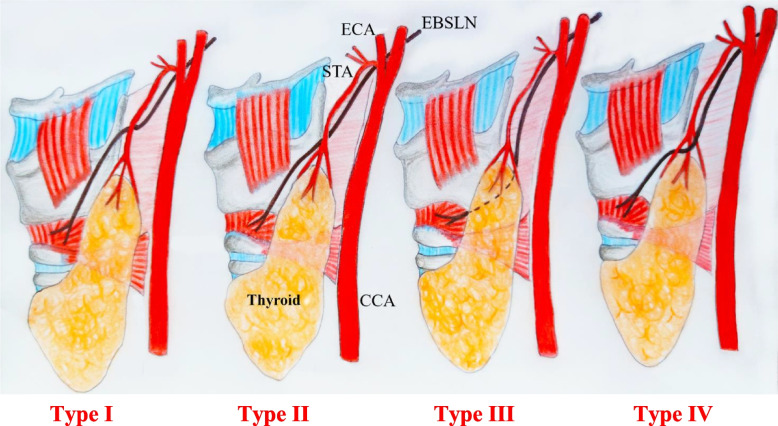
Classification of the EBSLN pathway according to Kierner. **Type I:** The artery-nerve crossing is more than 1 cm from the upper pole of the thyroid gland. **Type II:** The artery-nerve crossing is less than 1 cm above the upper pole of the thyroid gland. **Type III:** The artery-nerve crossing is above the upper pole of the thyroid gland. **Type IV:** The EBSLN descends dorsally and crosses the branches of the STA immediately above the upper pole of the thyroid gland

**Fig. 4:**
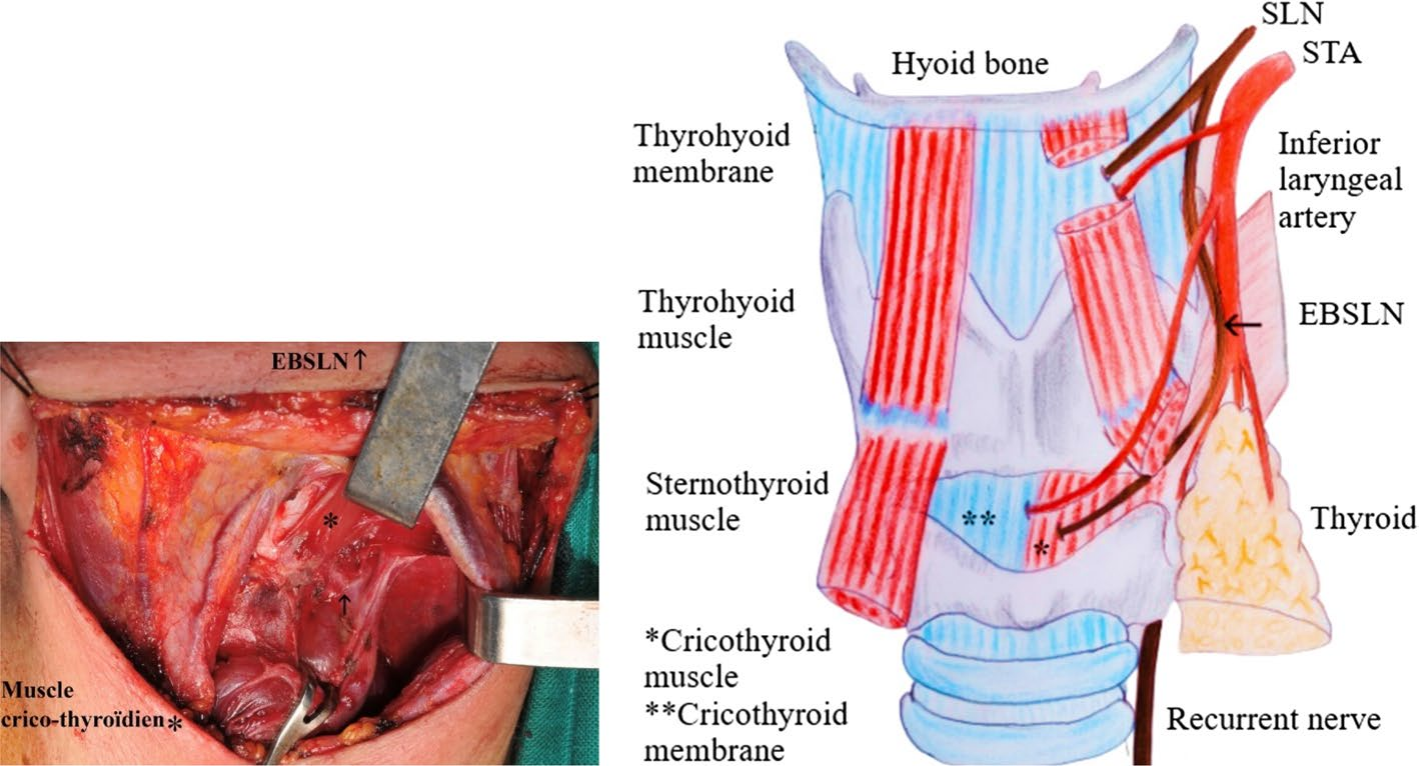
Type IIb Cernea and III Kierner where the nerve runs below the upper pole of the thyroid

**Fig. 5:**
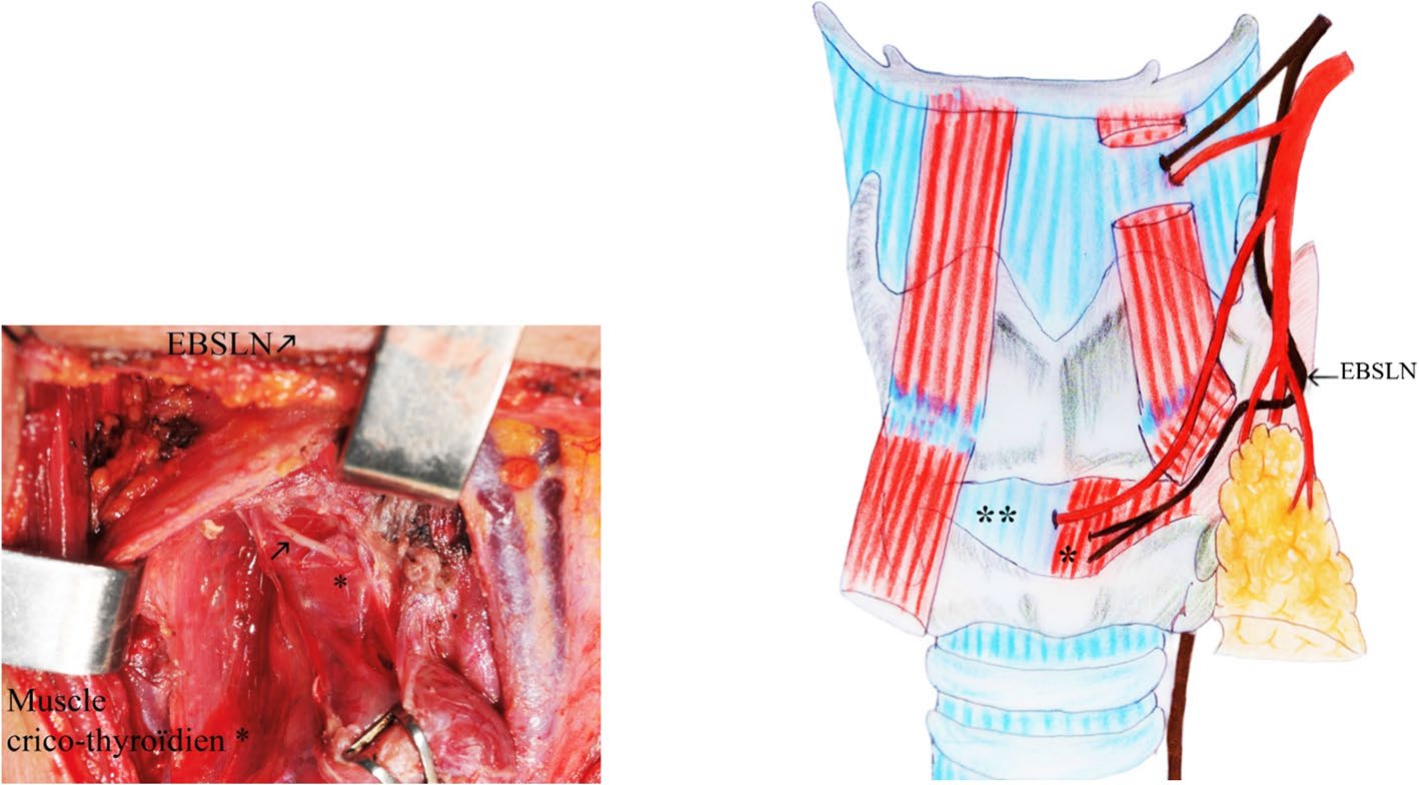
Type IV Kierner where the external laryngeal nerve passes between the trifurcation branches of the STA

Subsequently, Kierner [19] proposed a classification that integrates the anatomical types outlined by Cernea, adding a fourth type where the nerve passes through the trifurcation branches of the STA (Figs. 3, 4 and 5). This anatomical type exposes the nerve during dissection and especially during ligation of STA divisions.

All patients were examined postoperatively; a laryngoscopy was performed 15 days after surgery to assess the condition of vocal cords.

## Results

**Fig. 6:**
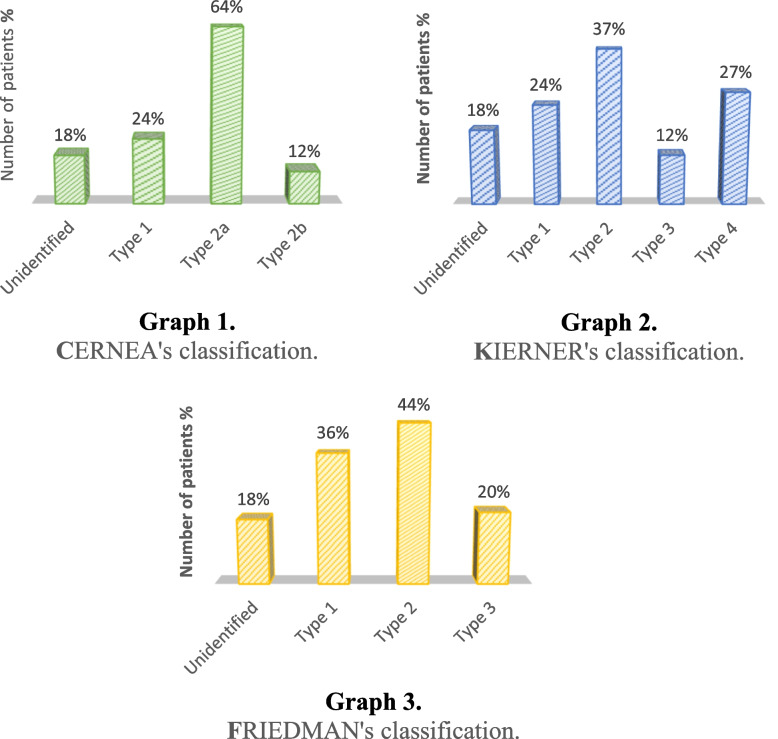
Distribution of nerves based on classification systems

The identification rate of the external laryngeal nerve in our serie was 82% (*n* = 41); It was not found in 18% of cases (*n* = 9), being type Ni. In 10% of cases (*n* = 5) the nerve was not found on the contralateral side. In the rest of patients, the type of nerve was similar on both sides. We classified the identified nerves according to different classifications. The EBSLN crossed the superior thyroid pedicle more than 1 cm from the upper pole of the thyroid (Type I Cernea and Kierner) in 24% of cases (*n* = 10), less than 1 cm (Type IIa Cernea) in 64% of cases (*n* = 26), and below the upper pole (Type IIb Cernea) in 12% of cases (*n* = 5) (Fig. 6: Graph 1). In 7 out of 26 type II Cernea patients, the EBSLN passed between the branches of the STA (Fig. 6: Graph 2).

The EBSLN remained superficial and wound along the surface of the IPC (Type I Friedmann) in 36% of cases (*n* = 15), crossed the muscle at the level of the thyroid cartilage (Type II Friedmann) in 44% of cases (*n* = 18), and traversed the muscle above the thyroid cartilage in 20% of cases (*n* = 8) (Fig. 6: Graph 3).

We noted no alterations in vocal quality after surgery, and the postoperative recovery proceeded without any complications. The laryngoscopy conducted 15 days following surgery yielded normal results for all patients.

Anatomopathological study revealed in 36% of our specimens a multi-heteronodular hyperplasia, 16% vesicular adenoma, 14% microscopic papillary carcinoma, 13% papillary carcinoma, 11% Graves’ disease, 4% lymphocytic thyroiditis, 2% microvesicular adenoma, 2% microvesicular adenoma, and 2% NIFTP.

## Discussion

In 1935 Amelita Galli-Curci, one of the most famous Italian opera singers of the twentieth century underwent a thyroidectomy to remove a large goiter. Subsequently, she experienced permanent alteration to her voice with an inability to reach the higher octaves required for her soprano arias, thus ending her career [20]. Damage to the superior laryngeal nerve, responsible for the lengthening and thinning of the vocal cords, was the suspected etiology.

**Fig. 7:**
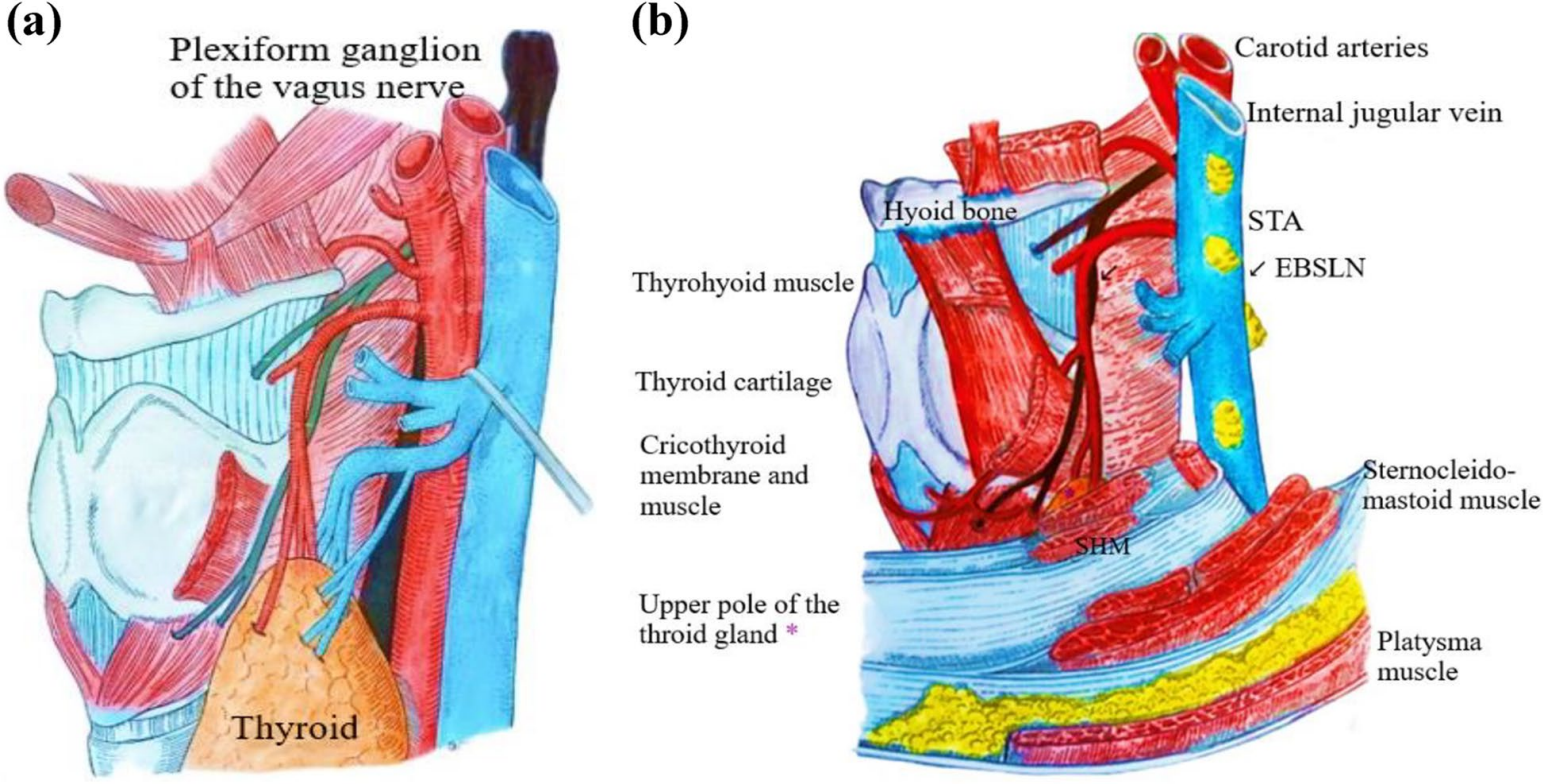
**a** Anatomy of the superior laryngeal nerve. **b** Connections of the EBSLN

Anatomically, The larynx is innervated by two main mixed nerves [21]; the SLN, which is mainly sensory, and the inferior (or recurrent) laryngeal nerve, which is mainly motor for the intrinsic muscles of the larynx. The SLN originates from the vagus nerve (VN), at the lower pole of the plexiform ganglion, at the level of the transverse process of C1 (Fig. 7a), witch is a good anatomical landmark in this regard. The SLN passes posterior to the internal carotid artery, through the superior cervical sympathetic ganglion and cervical sympathetic nerve chain, and then descends to the medial part of the thyrohyoid membrane [22]. Typically, the SLN is divided into internal and external branches in the superior angle of the hyoid [23]; the internal laryngeal nerve passes under the greater horn of the hyoid bone along the thyrohyoid membrane, and then crosses it through the same orifice as the superior laryngeal artery [24]. It provides sensory innervation to the glottis and laryngeal vestibule through the thyro-hyoid membrane. The external laryngeal nerve, which measures on average 6 cm, branches off from the superior laryngeal nerve 2-3 mm behind the greater horn of the hyoid bone. It describes a curve with antero-superior-internal concavity, and is divided into two segments with different directions, separated by an angle. In its first segment, the nerve has an oblique path downwards and forwards, deeply pressed against the pharyngeal wall. It then heads towards the larynx crossing the posterior edge of the sternothyroid muscle. On the external surface of the wing of the thyroid cartilage, inward, the nerve slides over the thyroid cartilage behind the insertion of the IPC. Outwardly, it contacts the STA and its branches (Fig. 7b). It is therefore potentially at risk during thyroid surgery [13]. The external laryngeal nerve may originate directly from the VN in a single trunk and independently of the superior laryngeal nerve in 5% of cases [25–27].

The current study reports an overall pooled EBSLN identification rate of 82%. Several studies reported similar identification rates [28–31], while others reported EBSLN identification rates ranging from 20% [32] to 100% [33, 34]. This heterogeneity in EBSLN identification rates could be attributed to topographical variations of the nerve, type of study (intraoperative or cadaveric), or the technique of nerve identification used (visual identification alone or with the aid of neuromonitoring devices).

Different nomenclatures based on the topography of the EBSLN and the correlated risk of injury have been proposed in large series. They suggest that the relationship between the nerve and the superior thyroid vessels is the most important surgical landmark that the surgeon must perceive to prevent injuries. Cernea et al. [16] proposed their nomenclature in 1992, and since then, it is the most widely recognized classification. The prevalence of Cernea type IIa and IIb represents 65% of cases in a meta-analysis of 24 autopsy and intraoperative studies conducted on 6098 dissected superior laryngeal nerves in subjects with normal and healthy thyroid and patients with thyroid pathology (Table 1).

**Table 1:** Prevalence of anatomical variations of the EBSLN according to Cernea classification

Subgroups	Number of studies (number of nerves)	Type I	Type IIa	Type IIb
All the groups	33 (6098)	39%	42%	19%
Peroperative	24 (5702)	35%	**44%**	**21%**
Cadaverous	9 (396)	50,5%	34,5%	15%

Our results yielded 44% Cernea type IIa, followed by type I and IIb. This result is similar to previous studies. Thus, type II represents anatomical forms at high risk, especially when the nerve is not sought and identified. There are differences in prevalence rates between anatomical studies on cadavers performed on subjects with normal and healthy thyroid and intraoperative studies performed on patients with generally hypertrophied pathological thyroid. Cernea reported significant differences in the prevalence rates of type I and II depending on the volume of the thyroid gland (Table 2). Type II represents 69% of cases for large goiters; this rate drops to 25% for small goiters. Kierner type II, III, and IV represent 58% of cases. The higher prevalence of type IIa and IIb nerves in intraoperative settings, particularly type IIb in patients with enlarged thyroid glands, should therefore be taken into consideration by surgeons during pre-operative planning of thyroid surgeries to facilitate intraoperative nerve identification, exposure and subsequent preservation, while minimizing its injury.

**Table 2:** Correlation of Cernea and Kierner classifications

Cernea [35]	Kierner [19]
Type I (68% SG, 23% LG)	Type I (42%)
Type IIa (11% SG, 15% LG)	Type II (30%)
Type IIb (14% SG, 54% LG)	Type III (14%)
Not described	Type IV (14%)

Friedman et al. [3] reported their experience in thyroid surgery in a retrospective study of 500 examined EBSLN (Table 3). We observe similar results, namely the difference between anatomical studies on cadavers and intraoperative studies. Type I is the anatomical form at high risk in intraoperative studies and represents 64% of cases, while type III nerves were the least common (20%).

**Table 3:** Prevalence of anatomical variations of the EBSLN according to Friedman classification

Subgroups	Number of studies (number of nerves)	Type I	Type II	Type III
All the groups	7 (500)	51%	34%	15%
Peroperative	3 (381)	**64%**	16%	20%
Cadaverous	4 (119)	37,5%	50%	12,5%

In our serie, Friedman type I represents 36% of cases, type II is the most dominant and represents 44% of cases, and type III represents 20% of cases. The classification by Friedman categorizes the EBSLN based on its relationship with the IPC [3, 36, 37]. Type I nerves are considered to be at the greatest risk of injury since it runs its entire course superficial to the IPC fibers [38]. Surgeons should be aware of the high prevalence of this type of nerves, particularly in the intraoperative setup. This may be helpful in carrying out safe dissection during thyroid surgery therefore minimizing EBSLN injury. Type III nerves on the other hand are considered to be the least susceptible to iatrogenic injury because it is protected by its intramuscular course through the fibers of the IPC [39]. Its course, however, limits its visual identification during surgery [40]. In fact, some authors consider EBSLN that could not be identified to be Friedman type III [34, 39, 40].

The connections of the EBSLN vary depending on the condition of the thyroid gland. When the thyroid is healthy and average-sized, the risk of nerve injury is relatively low, especially when the nerve runs at a distance from the STA and crosses it more than 1 cm from the superior pole of the thyroid gland. But when the thyroid is pathological, particularly in case of large goiter, the relationships are modified and the nerve can be located near the superior pole of the thyroid gland or even below this pole. In these cases, the risk of nerve injury during dissection of the superior pole of the thyroid gland is relatively high, particularly when it concerns an exposed anatomical type like type III Kierner, where the nerve runs below the superior pole of the thyroid, and even more so where the nerve is doubly exposed in type IV Kierner associated with type I Friedman.

The rate of identification and frequency of injuries of the EBSLN have been extensively studied. The technique for nerve identification varies among authors, ranging from surgical dissection alone to neuromonitoring of the external laryngeal nerve. Cernea reported the results of a comparative study of nerve identification using monitoring versus dissection (Table 4). It is interesting to note that the nerve identification rate is higher than 90%, and nerve identification drastically reduces the risk of nerve injury. In addition, the nerve injury rate is 0% when identification is performed by a senior surgeon, compared to 12% when the senior surgeon does not search for the nerve, and 28% if the patient is operated on by a resident.

**Table 4:** Rate of identification and injury of the recurrent laryngeal nerve during thyroidectomy

Autors	Evaluation methods	Nerve identification technique	ID %	Identification rate of the EBSLN %
Jonas [41]	Voice, laryngoscopy	Neuromonitoring	37,8%	4,6% transient
Lore [10]	Voice, laryngoscopy	No identification	33%	7,5% permanent
Teitelbaum [42]	Voice, EMG, videostroboscopy	Dissection on a case-by-case basis	-	5% permanent
Lennquist [43]	Voice, laryngoscopy	Inspection of the constrictor muscle without dissection	72%	2,6% permanent
Cernea [2]	Voice, EMG	Neuromonitoring versus dissection	93%	0% for seniors with nerve identification, 12% for seniors without nerve identification, 28% for residents without nerve identification, and 58% for transient cases.
Lebacos [44]	Laryngoscopy	Separate ligation of the STA branches without identification of the nerve	-	5.6% for high ligation, 0% for low ligation.

Lebacos [44] compared the results of high and low ultraligation of the division branches of the STA in terms of frequency of nerve lesions; 5.6% of permanent lesions were found with high ultraligation, and 0% with low ultraligation.

The EBSLN is particularly vulnerable during dissection of the upper pole of the thyroid gland. Ligating the STA exposes this nerve in Cernea type IIa and IIb, as well as in Friedman type I, and even more so in type IV described by Kierner.

**Fig. 8:**
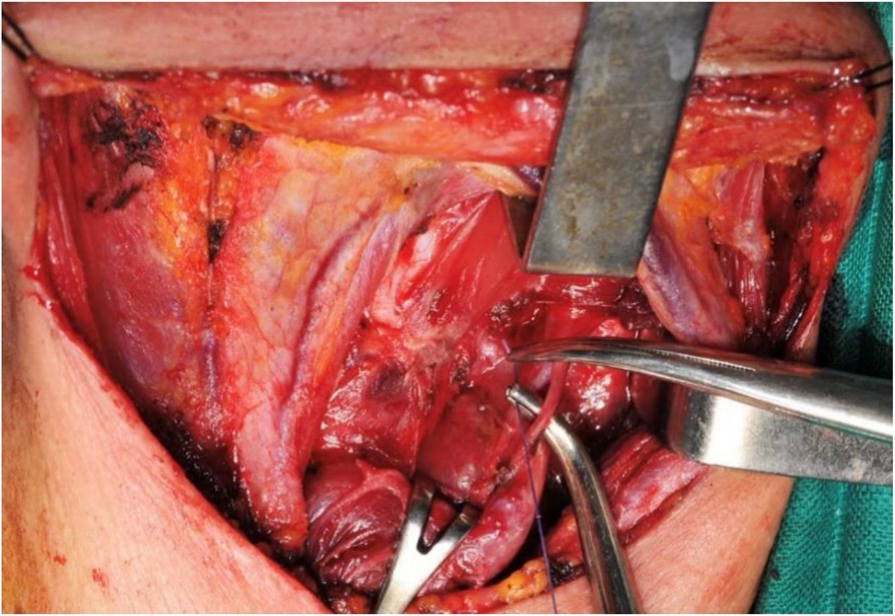
Elective ultraligation of the superior thyroid artery divisions

Adherence to good practice rules in thyroid surgery reduces the risk of injury to the superior laryngeal nerve and optimally preserves vocal functions. The first rule is a thorough understanding of the surgical anatomy of the EBSLN and variations in the laryngeal nerves. The surgical approach should balance aesthetics and safety; while a small incision may be aesthetically pleasing, it potentially exposes the EBSLN to risk due to inadequate and dangerous dissection. The dissection technique should be anatomical and bloodless and hemostasis procedures should be used rationally while respecting safety margin of each instrument. Identification of laryngeal nerves is necessary for optimal vocal functions safety and the search and dissection of the superior laryngeal nerve should be preferred over simple ultraligation without nerve identification. Intraoperative monitoring of the EBSLN facilitates nerve identification but does not prevent the risk of injury. Finally, elective ultraligation of the STA division branches should be performed as close to the thyroid gland as possible (Fig. 8) while preserving the posterior branch if possible. Mass ligation of the superior thyroid pedicle should be avoided.

## Conclusion

Anatomical variability of the EBSLN is again evident, highlighting the necessity for careful and knowledgeable surgical technique. The anatomical types found in this study were mainly Cernea type IIa and IIb, witch expose patients to a higher risk of injury during thyroidectomy. However, it remains possible to increase the identification rate of the nerve and avoid its injury even without employing sophisticated methods such as intraoperative neuromonitoring.

Thyroid gland dissection requires expertise to preserve the EBSLN. Large volume glands pose a more difficult challenge, as the gland is closer to the nerve. The external laryngeal nerve should be identified and preserved before ligating vessels at the superior thyroid pole. Additionally, cautious use of electrocautery devices is essential to prevent inadvertent damage, while also avoiding undue stretching of vascular structures, thereby mitigating the risk of iatrogenic injury.

## Data Availability

The datasets used during the current study available from the corresponding author on reasonable request.

## Abbreviations

CCA: Common Carotid Artery
CTM: Cricothyroid Muscle
EBSLN: External Branch of the Superior Laryngeal Nerve
ECA: External Carotid Artery
IPC: Inferior Pharyngeal Constrictor muscle
LG: Large Goiter
Ni: Non-Identified
NIFTP: Non-Invasive Follicular Thyroid Neoplasm
SG: Small Goiter
SLN: Superior Laryngeal Nerve
STA: Superior Thyroid Artery
VS: Vagus Nerve.
